# Towards the Development of Portable and In Situ Optical Devices for Detection of Micro-and Nanoplastics in Water: A Review on the Current Status

**DOI:** 10.3390/polym13050730

**Published:** 2021-02-27

**Authors:** Benjamin O. Asamoah, Emilia Uurasjärvi, Jukka Räty, Arto Koistinen, Matthieu Roussey, Kai-Erik Peiponen

**Affiliations:** 1 Department of Physics and Mathematics, University of Eastern Finland, P.O. Box 111, FI-80101 Joensuu, Finland; matthieu.roussey@uef.fi (M.R); kai.peiponen@uef.fi (K.-E.P.); 2 SIB Labs, University of Eastern Finland, P.O. Box 1627, 70211 Kuopio, Finland; emilia.uurasjarvi@uef.fi (E.U.); arto.koistinen@uef.fi (A.K.); 3 MITY, University of Oulu, Technology Park, P.O. BOX 127, FI-87400 Kajaani, Finland; jukka.raty@oulu.fi

**Keywords:** micro and nanoplastics, freshwater, sludge, optical detection, portable devices, in situ detection

## Abstract

The prevalent nature of micro and nanoplastics (MP/NPs) on environmental pollution and health-related issues has led to the development of various methods, usually based on Fourier-transform infrared (FTIR) and Raman spectroscopies, for their detection. Unfortunately, most of the developed techniques are laboratory-based with little focus on in situ detection of MPs. In this review, we aim to give an up-to-date report on the different optical measurement methods that have been exploited in the screening of MPs isolated from their natural environments, such as water. The progress and the potential of portable optical sensors for field studies of MPs are described, including remote sensing methods. We also propose other optical methods to be considered for the development of potential in situ integrated optical devices for continuous detection of MPs and NPs. Integrated optical solutions are especially necessary for the development of robust portable and in situ optical sensors for the quantitative detection and classification of water-based MPs.

## 1. Introduction

During the past decade, the concern with plastic litter has become a hot topic, both in science and in everyday life [1]. The alarming information on the fragmentation of plastics into smaller particles, e.g., microplastics (MPs; size range of 1 μm–5 mm) and nanoplastics (NPs; 1 nm–100 nm), is a current issue of debate because they can have adverse environmental and health effects [2,3]. Ongoing research [4] on MPs and NPs tries to understand the properties of these particles in the aquatic environment [5,6]. However, one thing is sure: plastic litter in aquatic environments is decaying into smaller particles [7] due to factors such as ultraviolet (UV) light from sunshine and mechanical abrasion [8,9,10] of both floating and sunken plastic litter. Mechanical abrasion can result from the interaction of plastics with other solid media, such as sand, with water, and wind.

Effort has been put into recycling metals, glass, cardboard, and paper, especially in developed countries. This increase in recycling is due to compliance with the related strict regulations and policies. Unfortunately, due to the current lack of fully efficient suggestions or protocols for managing and recycling, many different kinds of human-induced plastic litter remain untreated. These plastics disperse into natural water bodies, soil, and the atmosphere [11,12,13]. The presence of plastics in water bodies contributes to raising their toxicity levels. For example, recycled food packing and preservation plastics, although reducing plastic waste, are a potential source for increased toxic chemical additives, which can be released into food or water bodies [14]. The direct and indirect impact of MPs on the environment is now clearly acknowledged. For instance, the interdependence of climate change and microplastics has been recently demonstrated [15]. In reference to climate change, one can talk about the changes in water bodies as a result of the time-dependent increase of MPs and NPs pollution and resuspension, and their cyclical effect on the climate.

MPs are usually characterized in the laboratory rather than in the field. After sampling, they are typically separated from the sample matrix, which can contain other organic and inorganic matter. Then, they are characterized using various types of sophisticated chemical analysis methods [16]. The characterization aims to obtain information not only on the plastic type but also on the particle sizes and concentrations of MPs in environmental samples [17].

Currently, in situ detection and quantification of MPs is difficult or even impossible, because of a lack of applicable methods. Some environments, such as wastewater treatment plants and water-related industrial processes, contain high amounts of organic and inorganic solids, making in situ detection of less abundant MPs complicated compared with other materials, a challenge without sample pretreatments. Moreover, factors such as temperature and pressure variations in natural water bodies and (frozen) lakes complicate the problem. The variations in these parameters impact the local conditions at different heights (e.g., frozen top and unfrozen bottom and top layers) in the water bodies, which consequently affects differently the properties of the local MPs.

Among the different detection methods to meet the harsh and varying measurement conditions, photonics-based sensor solutions are promising in achieving the desired real-time data acquisition from MPs in situ. Demonstrating its potential, many of the existing MP identification methods in the laboratory already utilize photonics. Methods based on Fourier transform infrared spectroscopy (FTIR) and Raman spectroscopy [18,19,20] are examples. The advantages of photonics are based on the feasibility of non-destructive, label-free, real-time, robust, and inexpensive sensors. Photonics-based methods enable the determination of intrinsic and extrinsic properties of materials. For example, surface roughness, curvature, and transparency of plastics [21,22,23,24,25] can be determined simultaneously with its chemical compositions. These advantages make photonics-based solutions compatible with the detection and discrimination of very complex microplastic types originating from common synthetic plastics such as polyethylene (PE), polypropylene (PP), polyethylene terephthalate (PET), etc. MPs originating from tire wear, however, are hardly identifiable by popular photonics-based methods without further treatment of the samples [26]. Although independent photonics-based solutions, as demonstrated, present a powerful tool for the fight against MPs detection and analysis, their potential as an integrated solution of the various techniques for a robust and portable device for in situ detection of MPs in aquatic environments are yet to be explored due to the complexity of the problem, ipso facto, to be addressed.

In this review, we first briefly consider the physical phenomena behind light and plastic interactions, and the current status of photonics-based spectroscopic methods of MPs as well as their advantages and disadvantages. In particular, we consider the working principles behind the two widely used techniques—FTIR and Raman spectroscopies—to aid the understanding of non-photonics experts. We also consider possible portable optical devices with the potential to detect MPs in field conditions. Moreover, we further propose the development of photonics-based integrated sensors for in situ detection of problematic NPs in water. Finally, we provide hints on how integrated optical systems can be used to enable the development of portable devices for the detection of NPs in the natural complex environment. From these perspectives, we point out the gap between the many current laboratory-based MP- and NP-related studies and the requirements for in situ detection. We also propose and indicate the need for an integrated photonics-based solution towards the development of portable and in situ optical sensors in the fight against aquatic-based MPs. Although multifunctional photonics-based solutions, as proposed, are unavoidable in the tackling of MP identification, we note that specific applications such as in aquaculture will require further considerations in the combination of specific and applicable photonics solutions.

## 2. Important Optical Properties of Microplastics

Plastic is a general term for the description of synthetic and semi-synthetic materials that are produced from raw materials such as crude oil, coal, cellulose, etc., and are moldable into desired shapes and sizes. Due to their versatility, plastics have a wide range of applications in many areas including packaging, automobile, healthcare, and energy. Plastics can either be classified as thermoplastic—softens upon heating—or thermoset—solidifies when heated—in their response to heat. Our focus is on common and abundant MP and NP pollutants such as PET and PP, which are thermoplastics. These microplastics, with size < 5 mm, can be produced in small sizes (primary sources) or originate from the fragmentation of larger plastics (secondary sources).

**Figure 1:**
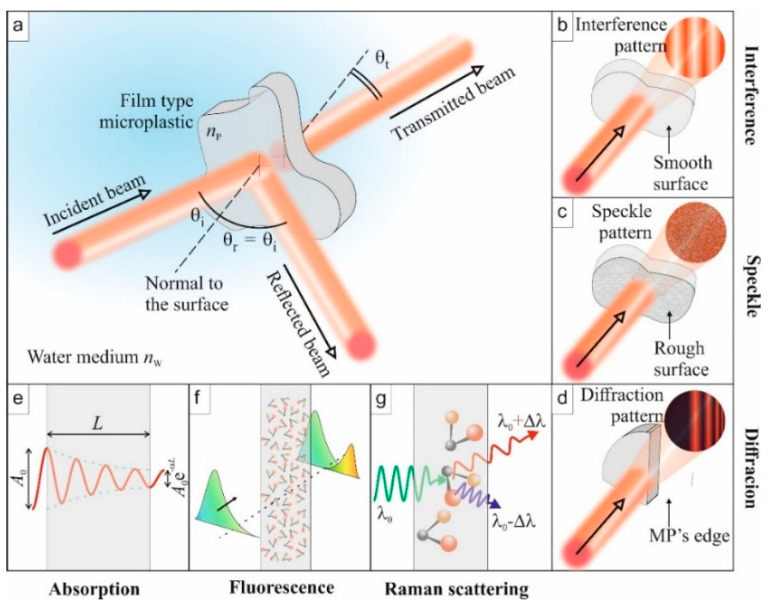
Illustration of some optical phenomena to be exploited for the identification of microplastic (MP) properties. (**a**) Overview of a standard experiment: the incident light is reflected, transmitted, and absorbed. (**b**) Interference: If the MP has two smooth surfaces, multiple reflections inside the film lead to an interferogram. (**c**) Speckle: Rough surfaces will generate a speckle originating from multiple interferences. (**d**) Diffraction: edges of the MP particles can diffract light beams to create organized patterns. (**e**) Absorption: the output amplitude is lower than the incident one. (**f**) Fluorescence: absorbed light energy is re-emitted at other wavelengths when molecules relax. (**g**) Raman scattering: specific light frequencies excite vibrational states of molecules, which lead to the emission of secondary photons at a slightly shifted frequency (Stokes and anti-Stokes).

For specified applications, plastic materials can be transparent, opaque, or colored. Similar to other materials, when plastics interact with electromagnetic (EM) radiation (light waves), irrespective of the incident wavelength of the light wave, some optical phenomena can occur, as illustrated in Figure 1. These phenomena are reflection (Figure 1a), refraction, absorption (Figure 1e), transmission (Figure 1a), and interference of light (Figure 1b). Upon the interaction of the two—light waves and the plastic—the plastic particle affects the properties of the emitted light wave. For example, the polarization of the electric field of the light wave can be modified, which can be detected by interferometry. The strength of the phenomena, ipso facto, depends on the intrinsic optical properties of the plastic and the wavelength of the incident radiation. The intrinsic optical properties are described by the index of refraction and the absorption coefficient of the medium, which manifests in the optical spectra. Reflection and transmission of the light wave, for example, constitute measurement methods of optical spectra such as Fourier transform infrared (FTIR). Additionally, excitation of MPs with suitably higher energy light waves can lead to subsequent light wave emissions at longer wavelengths. On the other hand, specific light frequencies can also excite the vibrational modes of the molecular constituents of MPs, leading to scattered secondary photons with slightly shifted frequencies.

In addition to the intrinsic optical properties, the geometry of MP particles, when compared to the wavelength of the probing light wave, can influence its optical response (spectra). In such a case, in addition to the five optical phenomena mentioned above, scattering of the light wave can occur. Considering the simple case of spherical primary MPs, classical Mie scattering theory can be used to analyze them. In reality, however, secondary MPs are more typical in aquatic environments and may take complex shapes, including fibers, films, sponges, and fragments [27,28,29].

In such a case, there is no general theory to describe the scattering of a light wave from the complex-shaped MPs. However, MPs can also diffract light waves from the edges (Figure 1d), which may provide information on the complex structure of the MP. Moreover, although the shape of an MP may be complex, it will exhibit its intrinsic optical properties, such as the index of refraction. The exhibition of intrinsic optical properties also holds for an MP with, due to mechanical wear, a rough surface [24]. Surface roughness is another factor, in addition to the size of an MP, that can affect the strength of light wave scattering [24]. Interacting with a coherent light source, surface roughness leads to the formation of a grainy pattern, called speckles (Figure 1c), that results from the random phase arising from the variation in local surface heights [30]. Below, we also introduce, in the context of Raman spectroscopy, another type of scattering mechanism of the probe wave.

Regarding in situ detection, the natural environment of MPs can also impact their optical characteristics. For example, MPs may adsorb contaminants that can be organic, such as bacteria and viruses, or inorganic, like metals [31,32]. This additional micro-or nanofilm, forming eco-corona, modifies the effective optical properties of MPs. The effect of these contaminants can also be optically detected, however not in situ [33].

In the case of aquatic-based NPs, plastics particles with at least one of the dimensions between 1 and 100 nm, other factors such as nano-roughness [34], and the properties of the surrounding matrix can affect the optical response. Unfortunately, current NP detection studies only focus on the commercial nanosphere NPs under controlled laboratory conditions, which differ significantly from the real environmental samples that can be polydispersed and polymorphic [35]. Indeed, for similar MPs harbored in freshwater and saltwater, the samples under the two conditions have shown different or similar optical responses under certain optical characterizations, indicating the need for complementary methods for the realistic detection of MPs and NPs in situ [33].

Similar to the geometry of the MPs, when NPs or their aggregates have a size much smaller than the probe wavelength of the light wave, Rayleigh scattering occurs, which is proportional to the inverse of the fourth power of the wavelength of the incident light wave, whereas Mie scattering dominates when the size is comparable or larger than the wavelength of the probe light wave.

Thus, optical spectroscopies in the frame of the linear optical process, such as reflection, absorption, and transmission of probe light waves, have poor sensitivity to the low number density of NPs and become erroneous in near-infrared (NIR) due to strong absorption of water. Additionally, for very small NPs, spontaneous scattering is very weak, and hence it is difficult to separate inelastically scattered laser light from intense Rayleigh scattering. Thus, efficient measurement methods, to be described in a later section of the paper, unlike the traditional FTIR and Raman spectroscopies, are necessary for better sensitivity and reliability for in situ detection of NPs in complex aquatic environments.

## 3. Spectroscopic Identification of Microplastics

Spectroscopy is the study of wavelength-dependent light–matter interactions, which has a long history in the characterization of materials, after its discovery. In this section, we examine the different spectroscopic methods used in the identification and characterization of MPs. Being the two most widely used spectroscopic techniques for MP identification, a somewhat detailed description of the process and device is given for Fourier-transform infrared and Raman techniques for readers who are new to the field.

### 3.1. Fourier Transform Infrared Spectroscopy

Fourier transform infrared spectroscopy (FTIR) is a vibrational spectroscopy, which identifies molecules based on how they absorb infrared (IR) light waves. In molecules, bonds between atoms vibrate in specific energy levels, and IR, interacting with the material, corresponding to this specific energy level are absorbed by the molecules. IR spectra, therefore, represent the absorbance as a function of the wavelength, typically expressed in wavenumbers (cm^−1^) where the spectral peaks correspond to specific bonds or multi-bonds of the studied chemical structures. As different molecules absorb IR differently, due to the different bonds present, FTIR spectroscopy presents a very powerful method for characterizing known and unknown organic compounds when compared with the spectral library. With its long history of polymer identification, FTIR has consequently gained popularity as an efficient method to study MPs [36]. Different versions of the FTIR devices, namely the transmission, reflection (specular and diffuse), and the attenuated total reflection (ATR)—exponential decay of intensity of evanescent wave—with their corresponding limitations, exist for different applications. In addition to its capabilities, the choice of the FTIR device is largely dependent on the size of the MPs.

The most affordable and easy to use instrumentation is ATR with a benchtop FTIR spectrometer. It has been used for the characterization of MPs, especially when studying larger particle sizes (>1 mm) or verifying the accuracy of other analytical methods, such as light microscopy [37,38,39]. However, ATR measurements are laborious, requiring a larger particle size for ease of handling and sample pretreatment. Moreover, surface modification of samples can interfere with the quality of the obtained signal [40], perhaps limiting the percentage confidence in identifying environmental MPs from spectra libraries.

To detect MP sizes down to approximately 10 μm, a relatively convenient option is to couple a benchtop FTIR spectrometer with a microscope. The configuration employs one of two detectors, namely a single-pixel or so-called point detector, which measures one spectrum at a time [41,42,43], and an imaging focal plane array (FPA) detector consisting of a square array of pixels, where each pixel measures one spectrum at a time [44,45,46]. Practically, a point detector requires manual or automatic [47] pre-selection of particles to measure, whereas FPA is used for larger measurement areas. Although both options of imaging-FTIR allow for the identification of smaller MPs, the efficiency of the pretreatment or separation from the sample matrix becomes very important [16], as inefficient processing leads to increased measurement time in the point detector. On the contrary, the presence of other materials hinders the spectral identification in FPA imaging.

**Figure 2:**
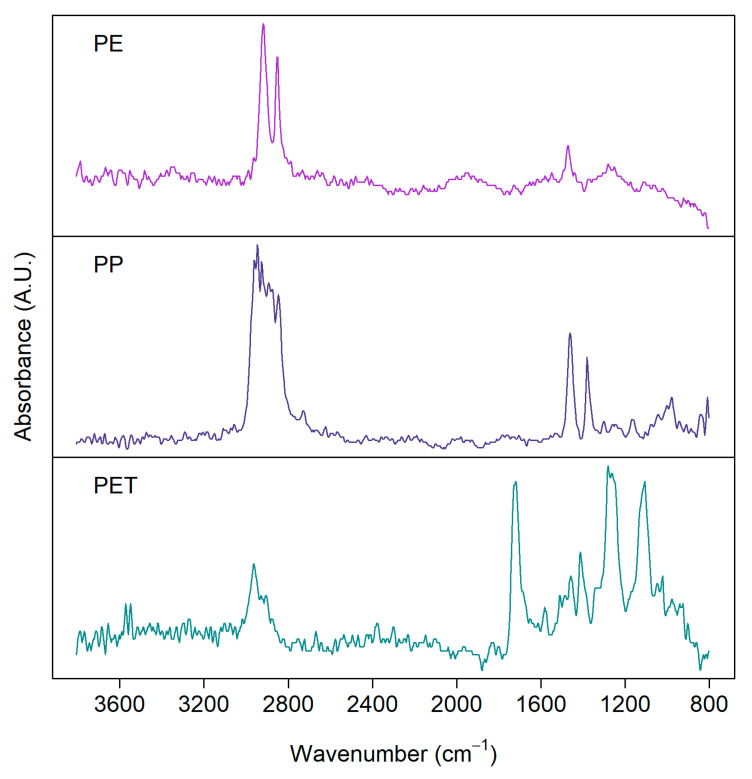
FTIR spectra of microplastics detected from environmental samples: polyethylene (PE), polypropylene (PP), polyethylene terephthalate (PET). Samples were measured with a focal plane array (FPA) detector in reflection mode from silver membrane filters.

Imaging-FTIR can be operated in reflection or transmission mode [48]. Generally, the transmission mode is more suitable for smaller particles, as larger and thicker particles heavily absorb the incident radiation. Reflection mode, on the other hand, suffers from variations in morphologies [49]. Figure 2 shows examples of FTIR spectra measured with the FPA detector in reflection mode for some common MP samples [50] from a real environment separated from biota samples showing clear distinction among the spectra for PE, PP, and PET, especially at higher wavenumber.

Although FTIR methods provide quantitative information, the classification of the spectra obtained from environmental MPs can be challenging. FTIR-obtained spectral data is usually analyzed by comparing sample and reference spectra [51]. The reference spectra can be commercial, custom-made, or free plastic libraries, including spectra of virgin and/or weathered plastics and MPs [52]. As the sample spectra usually deviate from the characteristics of the collected reference spectra, human interference is needed for classification. This assistance can introduce some bias [53]. Whilst single spectra, despite being time-consuming [53], can be manually searched against spectral libraries, classifying FTIR images comprising millions of spectra is challenging. Therefore, (semi)automatic data analysis methods and software have been developed for quantifying MPs from imaging-FTIR data [54,55,56,57]. The Match rate of reference and sample spectra is usually calculated as Pearson’s correlation coefficient, but also other algorithms such as hierarchical cluster analysis [51] and curve-fitting Python code [58,59] have been developed for MP research, significantly improving the classification. To improve the efficiency of the data analysis, spectra can be preprocessed to remove noise, correct baseline, and/or normalize the spectra [60].

Imaging-FTIR with an FPA detector has been proposed to be the standard method for quantifying small MPs from environmental samples [44]. Followed by automatic data analysis methods, it provides the counts, sizes, plastic types, and mass estimations of MPs in samples [54,57]. However, imaging-FTIR has disadvantages: the instrumentation is expensive and it requires special expertise. Additionally, spectral imaging of large areas is time-consuming and produces a large data set, which is also time-consuming to analyze. The major advantage of imaging-FTIR compared to point measurements, however, is the higher degree of automation, which reduces the laboriousness and subjectivity of the analysis.

Various factors affect the quality of FTIR spectra. As mentioned above, the measurement mode can be either reflection or transmission, which dictates the type of substrate to be used. For example, silver membrane filters [54], gold-coated filters [46], or microscope reflection slides [61] are used for reflection measurements, whereas zinc selenide windows [57], aluminum oxide (Anodisc) filters [62], or silicon filters [20] are also used for transmission measurements. In both transmission and reflection modes, the substrate materials should be non-absorbing in the desired wavelength range. The more these prerequisites are met, the higher the integrity of the obtained spectra. In addition to the effects of the substrate, the physical structure of MPs can cause interference and diffraction, as discussed in Chapter 2, which hinders the quality of FTIR spectra. FTIR spectra are usually presented as an average of multiple scans, and the signal-to-noise ratio can be increased by increasing the number of scans. However, for large area-imaging, a compromise between data quality and the single measurement time should be considered [44] to achieve optimum results. Lastly, FTIR techniques are challenged by colored samples, and the presence of water as both conditions leads to increased IR absorption [53].

### 3.2. Raman Spectroscopy

Raman spectroscopy is also vibrational spectroscopy, similar to FTIR. However, the physical phenomena behind the two non-destructive methods are different. For example, (FT)IR is sensitive to the variation in the dipole moment, whereas Raman is sensitive to the polarizability of the molecule. While FTIR measures the absorbance of light waves, Raman measures the scattering of the incident light waves in the visible light region. Scattering of light occurs when a photon hits a molecule and excites the bonds to the so-called virtual vibrational states. When the bond returns to a lower vibrational energy state, a photon is emitted, and the emission wavelength and intensity are measured. The energy and wavelength difference between absorbed and emitted light is presented as a Raman shift (cm^−1^). Similar to the FTIR spectrum, the Raman spectrum has peaks in specific wavenumber positions corresponding to bonds in molecules. With a library of Raman spectra, one can identify molecules, such as polymers and other components of MPs [63]. In terms of sample preparation and data analysis, both FTIR and Raman measurements utilize the same methods [64] (see Section 3.1 for FTIR and data analysis).

A Raman spectrometer consists of a monochromatic laser, a grating filter for removing the unscattered Rayleigh radiation (scattered light wave with the same frequency as the incident), and a charge-coupled device (CCD) detector. Raman is inelastic scattering consisting of Stokes and anti-Stokes scattering depending on the initial state of the bond before excitation. Because the ground state (initial state for Stokes) is always more occupied than the first excited state (initial state for anti-Stokes), Stokes scattering is a more common phenomenon than anti-Stokes, and therefore more sensitive to measure. Figure 1g illustrates the Stokes shift and anti-Stokes shift related to the nonlinear optical scattering phenomenon.

For MP studies, Raman microscopy, a spectrometer coupled to a microscope [63], is a popular choice because it enables the characterization of small particles down to 1 μm, lower than the detection limit of FTIR [18], although sample pretreatment is difficult. Another advantage of Raman spectroscopy compared to FTIR is a wider spectral range, which reaches 50 cm^−1^, enabling the identification of inorganic compounds. Moreover, Raman can provide information about additive compounds of plastics, such as colorants and fillers, in addition to polymer types [65]. Contrary to FTIR, Raman is not sensitive to water, which makes it a feasible option for in situ measurements in water environments. Additionally, contrary to FPA in FTIR, Raman microscopes lack such fast detectors. Therefore, Raman mapping is conducted by measuring areas point by point, and it is generally more time-consuming than FTIR [65].

As opposed to the absorbance in FTIR measurement, Raman scattering (Figure 1f) is rather very weak—only about one photon in a million scatters. Therefore, relatively high laser power is required for an optimum signal, which, on the other hand, can destroy sensitive samples such as very small MP particles. Moreover, the laser light source can induce fluorescence, which causes a strong background in the spectrum and overlaps with the Raman peaks, a problem not encountered in FTIR, complicating the interpretation of the measured data. Laser-induced fluorescence can be avoided using an excitation wavelength in the NIR region of the electromagnetic light wave [66]. Other methods have also been proposed for addressing the fluorescence issue, for example, subtraction of the fluorescence background after simultaneous excitation with two laser light sources with slightly different wavelength [67,68]. Moreover, since fluorescence is a delayed optical response compared to Raman scattering, a short-pulsed laser with gated detectors provides an alternative means to separately collect the suitable Raman signal [69]. In addition to the laser source, fluorescence masking of the Raman signal also arises due to the presence of inorganic and organic materials [18,70] and pigments [71], either limiting signal acquisition or influencing peak position. However, these sources of interference can somewhat be solved by suitable sample pretreatments [72], which may also impose degradation to the samples [73,74]. Additionally, automatic fluorescence correction and improved sensitivity of Raman detectors can address the issue [18].

Besides the fluorescence described above, the mere presence of non-fluorescing materials can also impact the quality of the Raman signal [75]. Although FTIR and Raman are limited by the credibility of the reference library—consisting of spectra from pristine samples—Raman is less sensitive to weathered MPs.

**Figure 3:**
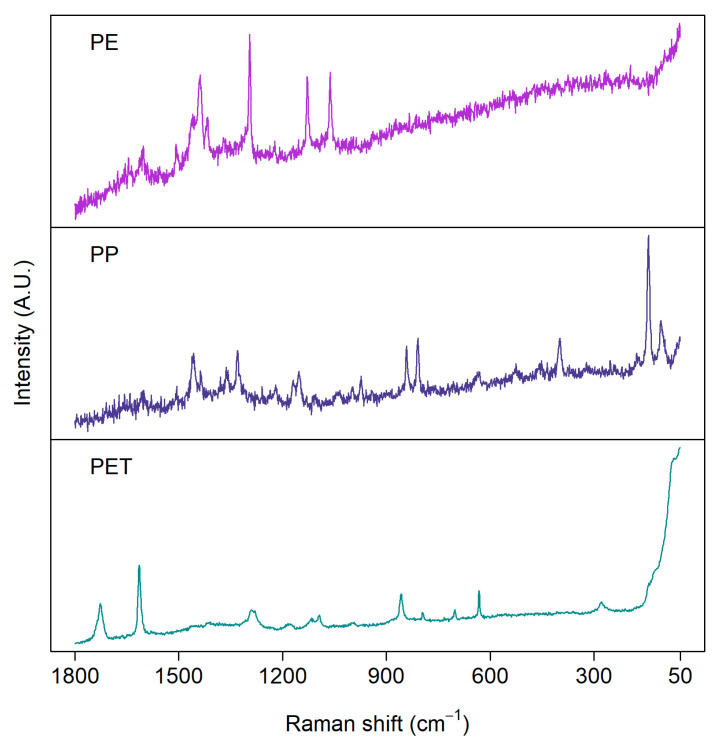
Examples of Raman spectra of microplastics measured from environmental samples: polyethylene (PE), polypropylene (PP), and polyethylene terephthalate (PET). Samples were measured with a 785 nm laser from aluminum oxide (Anodisc) filters.

Figure 3 presents examples of Raman spectra measured from the same set of real environmental MP samples shown for the FTIR measurements above. The quality of spectra varies between particles, although they have been measured with the same settings. Consequently, the same measurement parameters, such as focus, objective magnification, laser wavelength, and measurement time, are not suitable for every particle [65]. Thus, an optimal signal-to-noise ratio would require a long time to obtain a representative amount of particles analyzed. As in FTIR, the choice of the measurement parameters is always a trade-off between spectral quality and measurement time.

Raman spectroscopy has also been coupled with other techniques for the identification of NPs. For instance, instead of coupling to microscopy, it is rather coupled to optical tweezers [76], a technique demonstrated by Gilbert et al. [19]. The idea is to tightly focus a laser beam to trap a particle in the region of the focal point of the beam. Essentially, the particle is trapped by the strong electric field at the focal point, a phenomenon that was nicely demonstrated by Arthur Ashkin for which he was granted a Physics Nobel Prize in 2018. The force exerted by the focused light depends on the refractive index of the MP or the NP to be detected. The nice feature of Raman tweezers is that submicron (20 μm down to 50 nm) plastic particles can be identified. Moreover, the method can discriminate between plastic, organic, and mineral particles [19]. Furthermore, thanks to the microscope, information on the size and shape of both MPs and NPs can also be obtained. However, the challenge regarding in situ detection using Raman tweezer is that the force for trapping of a particle is very weak; the typical motion of water in natural water bodies and wastewater filtration systems can disturb the trapping of the MPs.

A well-established measurement method to detect micrometer-sized samples is based on surface-enhanced Raman scattering (SERS) [77]. This technique is based on a nanostructured metal substrate. The metal substrates typically used are silver and gold. The SERS signal in the proximity of the metal is enhanced by several orders of magnitude compared with the conventional Raman signal. The enhancement is due to the so-called plasmonic behavior of metallic nanostructures. Lv et al. [78] successfully exploited SERS in the monitoring of MPs. In a way, this method also requires sample preparation because MPs must be in the proximity of the metal structure. Although the signal from Raman spectroscopy is weaker compared to that of SERS, it has also been used for the detection of MPs in simulated natural water environments [79].

Other Raman-related methods used in the detection of MPs are stimulated Raman scattering microscopy [80] and coherent anti-Stokes Raman scattering microscopy (CARS) [81,82]. CARS belongs to the category of the so-called third-order nonlinear optical phenomena [83]. The CARS spectrum can be detected using two lasers simultaneously—a high-power laser for pumping of the medium, and a wavelength-tunable low-power laser for probing the medium to detect characteristic Raman spectra of the scatterers. However, due to the requirements of expensive instrumentations and expertise, these methods have not gained as wide popularity in MP research as traditional Raman and FTIR spectroscopies.

### 3.3. Transmission Spectroscopy

In addition to FTIR and Raman, NIR transmission spectroscopy has also been proposed for the identification of MPs in water under laboratory conditions [84]. Transmission spectroscopy, from the sample point of view, works similarly to molecular absorption (Figure 1e) of NIR light waves, leading to spectra that are rich in overlapping overtones and their combinations. However, from the device point of view, transmission spectroscopy is based on the conventional spectrophotometer, which is much slower than the FTIR that is based on Michelson interferometry.

**Figure 4:**
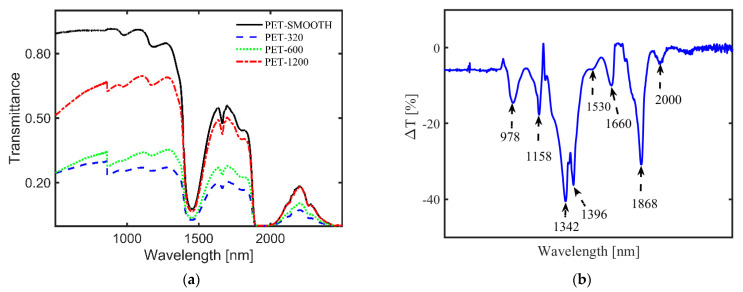
(**a**) Influence of surface roughness on the transmittance of film-type MPs, with a rough surface on both sides, in water [24]. MPs were prepared from polyethylene terephthalate (PET) plastic roughened with different sandpaper grits, 1200, 600, and 320, to achieve average surface roughness values of 0.34 μm, 0.60 μm, and 1.10 μm, respectively. The transmittance of the MPs nonlinearly decreases with increasing average surface roughness over the whole spectral range. (**b**) Difference in transmittance (ΔT) between the pure ethanol only and ethanol-containing MPs from a real sludge sample [submitted elsewhere]. It allows the immediate authentication of characteristic peaks of certain plastics in an extremely complex environment: polystyrene (PS, λ = 1158 nm), polyethylene (PE, λ = 1396 nm), and polyethylene terephthalate (PET, λ = 1660 nm).

Although not a fully developed identification method for MPs, like in the case of the other two methods, NIR transmission of MPs provides other useful information on MPs. For example, one can monitor the surface roughness and its time-dependent change, which can serve as a measure for the residence time of MPs in aquatic environments [85]. Moreover, transparent, translucent, and MPs with surface roughness can be differentiated from one another in water by measuring their transmittance [24,84]. Figure 4a illustrates an example of transmittance of thin-film PET samples with different roughness in water [24], showing a nonlinear dependence with the average surface roughness [22]. Additionally, for different MP types in a complex matrix, similar identification and classification based on spectra libraries, as in the case of FTIR and Raman, can be obtained via peak attribution of the weighted spectra. Figure 4b illustrates the difference in transmittance (ΔT) spectra for pure ethanol and ethanol with different environmental MPs suspended in it (FEMPs), where some dips in the curve, namely 1158 nm, 1396 nm, and 1660 nm, have been attributed to the PS, PE, and PET, respectively, in comparison to the studies in [84,86]. Thus, the difference in transmittance allows immediate authentication of characteristic peaks of certain plastics in an extremely complex environment, which are otherwise absent in the conventional transmittance of FEMPs. In the case of in situ detection of arbitrary MPs or mesoplastics in water, analytical tools such as Kramers–Kronig light dispersion relation can be convenient for the identification of the plastic type and surface properties [25]. Thus, the existence of a transmission spectra library of MPs under varied and complex conditions can prove very useful for the identification of aquatic MPs using this method. In particular, the use of classification techniques such as PCA [87] could be beneficial to MP identification.

Despite the potential of NIR transmission spectroscopy in aquatic MP detection, there exist some practical implementation challenges. In the NIR spectra range, water shows strong absorption of the light wave, which overshadows the spectral features of plastics present in the same spectral region. This effect of water absorption can, however, be avoided by decreasing the optical path length of the light radiation. Additionally, since some dense plastics such as polyoxymethylene (POM) have spectral features only in the mid-IR range [86], a much wider spectral range should be considered when using this method.

### 3.4. Fluorescence Spectroscopy Using Staining of Microplastics

Fluorescence spectroscopy (Figure 1f) is a very sensitive method to monitor the low concentration of typical organic materials. A medium that absorbs UV radiation may emit light at higher wavelengths, resulting in an emission spectrum that can be used for the identification of materials. Not all materials, however, show intrinsic fluorescence. Such materials can, therefore, be detected by labeling with a fluorescence tag.

When the other spectroscopic methods do not suffice, or visual counting preceding identification and characterization of MPs is challenging, fluorescence techniques are employed to enhance the process, as proposed by Andrady [88]. Such techniques, sometimes employing heat-assisted treatment, were successfully used to screen MPs by staining them with Nile Red [89,90,91,92,93,94]. It has, however, also been considered for NP identification [95]. Recently, Nel et al. [96] have also published a comprehensive study on sample preparation and identification of stained MPs to examine the detection limit when using Nile Red [96]. The use of Nile Red leads to improved selectivity of MPs compared to natural particles [93,97] and provides a relatively low-cost and faster identification [98]. Further, for weathered particles with irregularities, Nile Red fluorescent provides a relatively easier identification than with FTIR.

**Figure 5:**
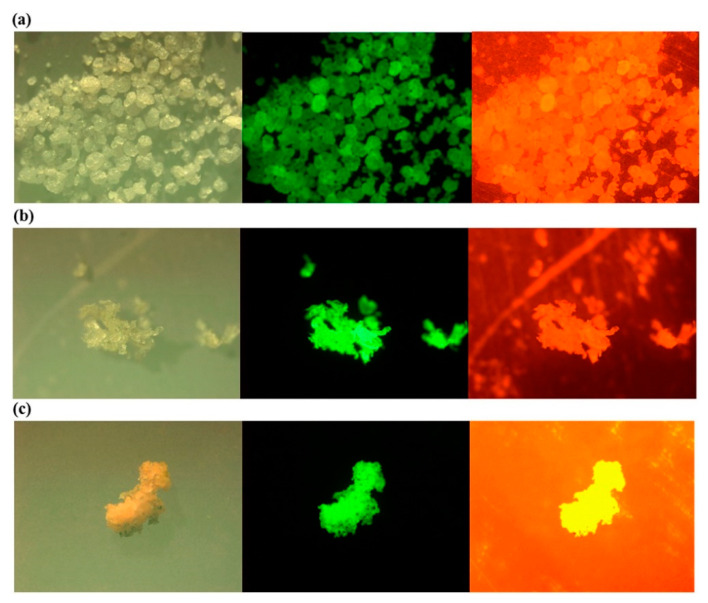
Photos under a microscope (left), a fluorescence microscope with excitation and emission wavelength of 534–558 and 515–565 nm (middle); and 534–558 and >590 nm (right) for the Nile Red stained low density (**a**) polyethylene (LDPE); (**b**) polypropylene (PP); (**c**) expanded polystyrene (EPS) Reprinted from *Marine Pollution Bulletin*, 113, Shim, W.J.; Song, Y.K.; Hong, S.H.; Jang, M., Identification and Quantification of Microplastics Using Nile Red Staining, 469–476. [93], Copyright (2016), with permission from Elsevier.

As with the other techniques, the detection of MPs by staining with Nile Red also has its challenges. For instance, there is the possibility of staining other natural particles, leading to unwanted emissions from these particles and the overestimating of MP concentrations [99]. To mitigate this effect, sample pretreatment and polymer-specific identification threshold should be considered [96]. Additionally, the effectiveness of Nile Red depends on its derivatives, concentration, and the type of solvent, and the excitation source and time [100] should also be considered. Red fluorescence of MPs, as seen in Figure 5 (right), contributes to the background staining, which complicates the actual MP identification, whereas green fluorescence is much more suitable [93,97]. Despite these challenges, a semi-automated method based on Nile Red tagging has been developed for the identification of smaller MPs [89,90], which, however, requires sample pretreatment.

### 3.5. Hyperspectral Imaging of Microplastics

Hyperspectral imaging (HI) is another well-established technique that has been exploited in remote air-borne sensing of natural objects such as forests [101]. The goal is to obtain both image and spectral information from an object under study. Different scanning methods for HI exist, namely point, line, and area scanning of samples. However, the core idea is to produce a typical output probe beam in a form of a line. To obtain image information, the line is scanned over the object by either moving the device or the object itself. Similar to the FPA–FTIR method, the detecting camera records the spectrum from each pixel within the defined spectral range, however either in the visible or NIR spectral range. Due to the simultaneous collection of both image and spectral data, it results in a huge data file with some redundant information. Therefore, to obtain useful information, further processing is usually needed. Huge data processing techniques based on supervised and unsupervised algorithms such as principal component analysis (PCA), support vector machine (SVM) in combination with PCA, and partial least squares discriminant analysis (PLS-DA) with PCA, respectively, are some of the popular classification methods [102,103,104].

**Figure 6:**
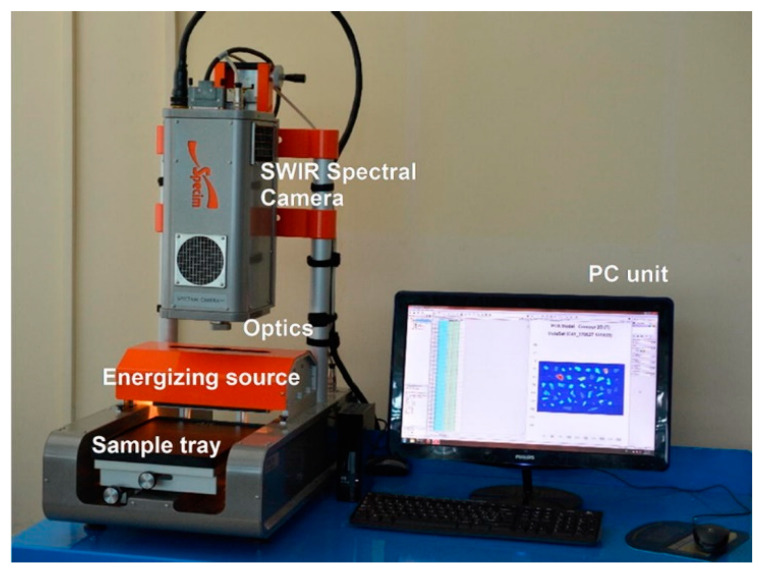
An overview of SISUChema XL™ Chemical Workstation (Specim, Finland) Reprinted from *Waste Management*, 76, Serranti, S.; Palmieri, R.; Bonifazi, G.; Cózar, A. Charac-terization of Microplastic Litter from Oceans by an Innovative Approach Based on Hyperspectral Imaging, 117–125. [104], Copyright (2020), with permission from Elsevier.

HI has been applied under both laboratory and field conditions. Figure 6 shows an image of a laboratory-based hyperspectral imaging system for MP identification. For field applications, HI has been used with unmanned aerial vehicles (UAV), e.g., drones, to monitor coastal MPs [105] and, in combination with photogrammetry, to map coastal transparent plastics such as bottles and bags, which are also primary sources of MPs [106]. The advantages and disadvantages of current MP identification methods, including hyperspectral imaging, have been described in a recent focused review [107]. The method is promising in the detection of reflection spectra from MPs and the provision of information on the size and surface area of a relatively large-size MP. It has also been proven to enable wide spectral range hyperspectral imaging of marine-harvested plastics. [108]. However, there are some issues of strong absorption by water, especially in the NIR range, when the MP is fully or partially covered in it. Additionally, the size of MP detection is currently limited to 100 μm [103] and, therefore, cannot detect NPs. Further, as with the FTIR, environmentally-modified samples show variations in recorded spectra, negatively influencing the accurate identification of MPs [102]. With regards to data processing, issues such as pre-processing of data are usually essential for efficient MP classification [109]. Lastly, since MP identification is based on reflectance spectra from HI, the absolute reflectance should be obtained using, for example, reference panels. However, this objective is hampered by changing the illumination conditions of the object arising from changing the cloudiness of the sky. Nonetheless, calibration issues of HI are well-described in [110].

## 4. Handheld Optical Devices

**Figure 7:**
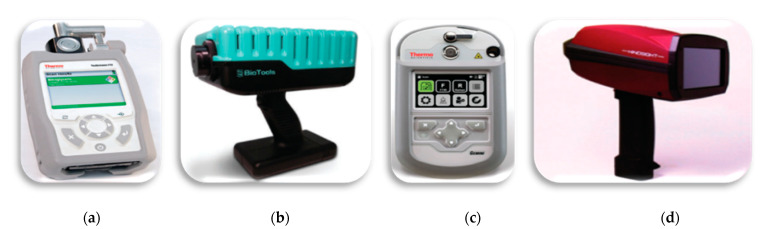
Portable devices: (**a**) FTIR—Thermo Fisher Scientific; (**b**) Raman—BioTools RamTest; (**c**) Gemini-combined Raman and FT-IR handheld analyzer—Thermo Fisher Scientific; (**d**) HI-BaySpec’s GoldenEye. Reprinted from *Applied Spectroscopy*, 72, Crocombe, R.A. Portable Spectroscopy, 1701–1751. [69], Copyright (2018), with permission from SAGE Publications

Thanks to the development of small size light sources, detectors, and optical elements and the need for field measurements, various types of portable and handheld commercial spectrometers are available. Some of these miniaturized systems are suitable for environmental monitoring issues [69]. Optical sensors belonging to the category of commercial portable and/or handheld devices are based on FTIR, Raman, their combinations, and HI [69,111] methods (see Figure 7).

FTIR- and Raman-based methods have found applications in raw materials quality inspection in pharmacy [112]. In addition to quality inspection, handheld FTIR and Raman methods can be used for the screening of counterfeit pharmaceutical tablets. Typically, the blister material is also made of plastic, through which the tablets are probed. FTIR portable spectrometers are also used for homeland security to detect dangerous gases and for remote monitoring of pollution due to exhaust gases from vehicles, chimneys of factories, and gas emissions from the dump. Gradually, these portable devices are gaining popularity in the detection of MPs.

Recently, a handheld FTIR spectrometer was used in the detection and identification of four different types of meso- and micro-sized plastics that were harvested in the coastal area of Greenland [113]. It is also probable that handheld Raman devices and multi-function handheld sensors, where both FTIR and Raman are coupled together, can be exploited in the monitoring of MPs from the environment. However, the analysis of MPs in situ with portable FTIR/Raman spectrometers is challenging because MP concentrations in the environment are typically low. Additionally, samples for FTIR measurements require the separation of MPs from the matrix before measurement. Moreover, because water absorbs IR, FTIR is less suitable for in situ monitoring of MPs in water environments. The methods for separation and detection of MPs in the field in situ will require major developments. Despite these challenges, Iri et al. [114] developed an inexpensive portable Raman sensor for the detection of micrometer-sized plastic coated magnetic spherical particles in water in a quartz cuvette.

**Figure 8:**
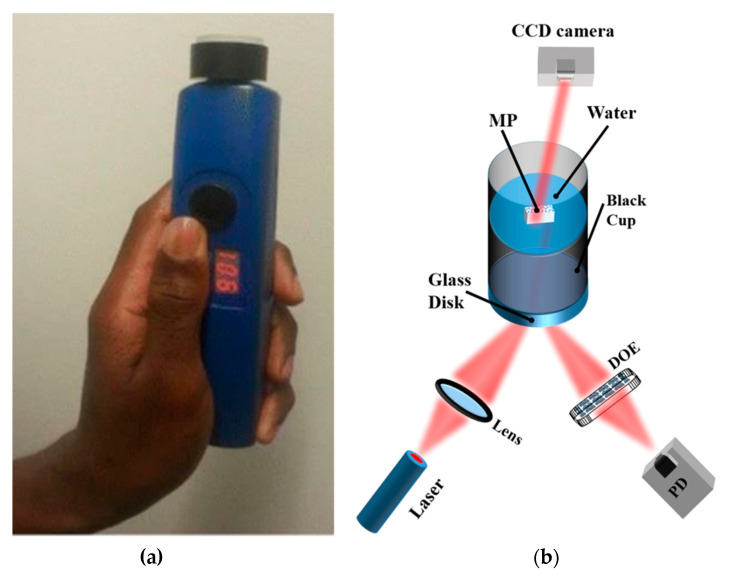
(**a**) Image of a handheld optical sensor for MP detection [117]; (**b**) optical measurement setup. The diffractive optical element (DOE) spatially filters the reflected light signal to obtain the specular component of the light signal on the photodiode (PD). The speckle pattern is measured by the CCD camera Reprinted from *Chemosphere*, 254, Asamoah, B.O.; Roussey, M.; Peiponen, K. On Optical Sensing of Surface Roughness of Flat and Curved Microplastics in Water, 126789. [23], Copyright (2020), with permission from Elsevier.

**Figure 9:**
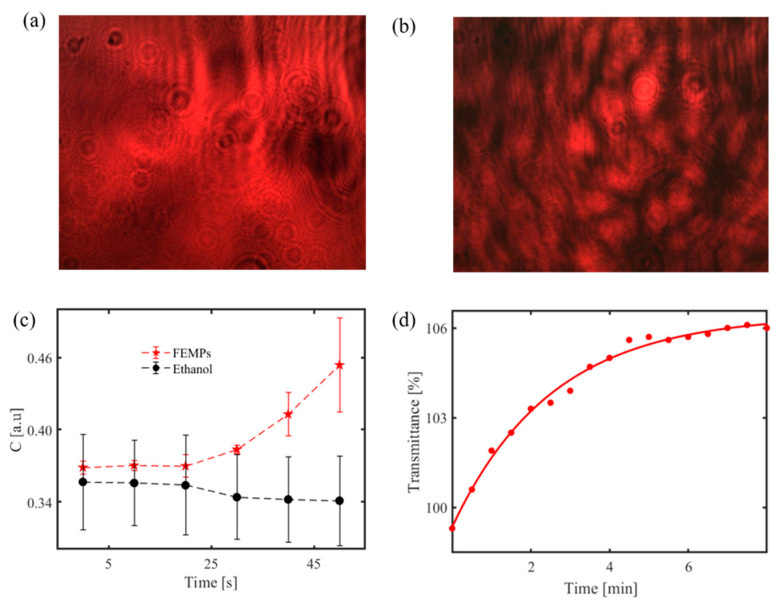
(**a**) Recorded interference pattern for transparent and smooth PET MP [21]; speckle pattern of similar MP with a rough surface [23]; (**b**) using the handheld optical sensor. Identification of MPs in ethanol; (**c**) time-dependent speckle contrast calculated for pure ethanol only and ethanol-containing MPs (FEMPs) on a rough glass disk using a handheld optical sensor; (**d**) time-dependent transmittance signal from ethanol containing MPs at a fixed wavelength (800 nm) using a spectrophotometer. Figure 9a Reprinted from *Chemosphere*, 231, Asamoah, B.O.; Kanyathare, B.; Roussey, M.; Peiponen, K.E. A Prototype of a Portable Optical Sensor for the Detection of Transparent and Translucent Microplastics in Freshwater, 161–167. [21], Copyright (2019), with permission from Elsevier; Figure 9b Reprinted from Chemosphere, 254, Asamoah, B.O.; Roussey, M.; Peiponen, K. On Optical Sensing of Surface Roughness of Flat and Curved Microplastics in Water, 126789. [23], Copyright (2020), with permission from Elsevier.

Recently, a prototype of a handheld optical sensor has been developed for the investigation of transparent and translucent a priori MPs (1–5 mm) [21,22,23]; see Figure 8. Unlike the more quantitative and matured spectroscopic devices like FTIR and Raman, this sensor monitors reflection, transmission, diffraction, and scattering of red laser light waves from MPs using a photodiode and a charge-coupled device (CCD) camera, without an imaging objective, as detectors. The sensor provides information on the smoothness and roughness, flatness/curviness, size, and sedimentation of MPs, parameters that are essential for in situ detection. Samples with surface roughness or volume inhomogeneity, due to porosity, scatter incident radiation of a coherent laser light source to form a speckle pattern on the CCD camera. Figure 9a,b show the interference and speckle patterns of transparent and smooth PET MPs and MPs with surface roughness, respectively, recorded with the handheld sensor. The speckles originate from the rough surface of the MP and the correlation of the magnitude of the surface roughness estimated from analyzing the grainy laser speckle pattern. Thus, the adherence of toxic and non-toxic materials, e.g., heavy metal compounds and organic materials [1,31,115,116], leading to surface modification of MPs can also be monitored. Below, we show some typical results of the device to indicate the scattering characteristics of MPs under different conditions.

The speckle pattern can also be projected to monitor moving MPs in water [118]. Figure 9c shows such a result from the handheld sensor. It illustrates the speckle contrast *C* variation with time, with error bars, calculated from the recorded speckle pattern for 700 μL of pure ethanol and ethanol-containing filtrated MPs (FEMPs). The speckle pattern is first generated by the interaction of the coherent laser source and a rough glass at the volume compartment to probe the liquid volume. When the liquid containing the MPs is introduced to the optical sensor, the particles modify the surface roughness, which correspondingly changes the speckle pattern on the CCD camera. By analyzing the speckle pattern to obtain the speckle contrast [30], one can monitor the time-dependent sedimentation of the MPs in ethanol, which is different from that of pure ethanol.

Although not from the portable device, Figure 9d also shows an exponential growth of transmittance with time for the FEMPs using a spectrophotometer to record the transmittance at a fixed wavelength of 800 nm. It suggests an increase in sedimentation of the particles increases with time, at least for particles across the beam, decreasing the amount of the scattering of the probing beam, and thereby increasing the transmittance. Similar to the handheld measurements, the cuvette containing the FEMPs was shaken before the measurements and probed midway up the cuvette height.

## 5. The Gap Between Current Detection and Preferred In Situ Detection

It is well acknowledged that MP and NP pollution is ubiquitous in our ecosystem, and a lot of effort, as described above, is therefore being channeled to address the issue from various fields. However, based on the several studies considered above, it appears that current identification methods are still limited to the laboratory, where sample type and size are a priori known, and measurement conditions are relatively stable. Thus far, only the HI technique in combination with UAV comes close to addressing the issue on the field or in situ. Even with this, it is still limited to the large plastics on the water surface [106], neglecting the in situ challenge. However, most abundant plastics in the environment (aquatic) are small (<500 μm) [89,119,120,121], requiring the development of more sophisticated techniques for in situ detection and the accurate estimation of MP/NP identification and concentration determination.

The reasons for the gap between current methodology and the required in situ detectors could be summarized as follows: rather than having dedicated solutions to address the issue, we are using conventional methods which are limited in many ways; as evident from MPs from the environment, these particles are usually different in morphology and further show different characteristics from their pristine counterparts. To have a perspective of the two reasons, let us briefly touch on the lifecycle of plastics in the aquatic environment.

It is evident that the plastics released into the environment (aquatic) are severely impacted by the habitat and therefore degrade further depending on their initial conditions. The various types of degradation are nicely summarized in [122]. The effects of these degradations are changes in the physical, optical, mechanical, chemical, etc. properties, leading to, for example, fragmentation, coloration, and variation in optical response [122]. Additionally, since MPs are interacting with other particles in the environment, it leads to the collection and accumulation of foreign materials, in which case the sample becomes heterogeneous. The consequence of these environmental interactions, further complicated by the harsh method of pretreatments, is the manifested variation in the optical response of environmental MPs to methods such as FTIR and HI.

Furthermore, the effect of the morphology and geometry of MPs on other non-spectroscopic techniques is also convoluted. In MP-related studies, information on the size/thickness, shape, refractive index, and concentration are also crucial; therefore, non-spectroscopic methods such as dynamic and multi-angle light scattering (DLS and MALs) [123,124], leading to speckle pattern formation, becomes essential. Such methods have been successfully demonstrated for mono-and poly-dispersive spherical particles under laboratory conditions. However, in situ, the presence of polydispersed MPs of different plastics types and other minerals and irregular organic materials can render this method unreliable. For example, Brownian motion of non-fiber-like MPs in water, and porous MPs with a high probability of multiple scattering, can complicate the scattered pattern, requiring rigorous analysis to extract useful information.

An additional limitation of the current identification techniques is the method of reference library creation and the classification methods. By far, most of the reference libraries are either largely based on pristine samples or are locally created. As we have seen, this approach poses a problem for the classification methods, limiting the accurate identification of MPs. Although fast and automated classifications are being developed in this regard, a consolidated library reference including spectra from different environments could prove beneficial to the integrity of MP identification and quantification, especially when it comes to in situ, paving way for the application of artificial intelligence.

Based on the summary above, it is therefore presupposed that to holistically develop optical sensors for in situ application, the above issues, in addition to the impact of a complex matrix such as water, should be considered, and the current optical methods need to be complemented. Fortunately, other solutions can be adapted. Thus, we consider some optical techniques that may be useful for the development of in situ detectors under the following categories:

1. general (Section 6) and
2. integrated solutions (Section 7).

## 6. Development of In Situ Detection Techniques for Screening of Microplastics and Nanoplastics

The best option regarding in situ real-time detection of MPs is to eliminate sample treatment. To achieve such a goal, there exist different possibilities. Although one cannot quantify the absolute results of these possibilities, their potential in the development of a sophisticated optical sensor for in situ application is unavoidable.

### 6.1. Fluorescence, Digital Holography, Dynamic Light Scattering, Optical Fibers, and Immersion Liquid Techniques

**Figure 10:**
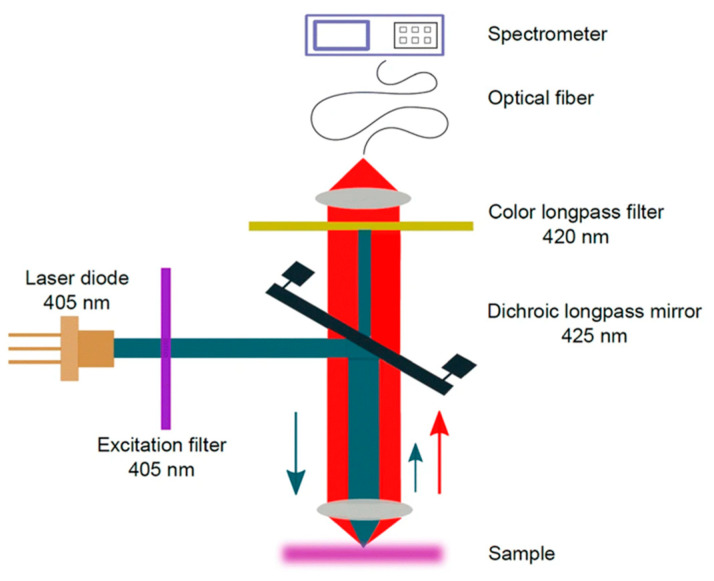
Scheme of the photoluminescence (PL) setup. The blue and red arrows indicate the propagation direction of laser and photoluminescence light, respectively Reprinted from Applied Physics B: Lasers and Optics, 126, Ornik, J.; Sommer, S.; Gies, S.; Weber, M.; Lott, C.; Balzer, J.C.; Koch, M. Could Photoluminescence Spectroscopy Be an Alternative Technique for the Detection of Microplastics? First Experiments Using a 405 Nm Laser for Excitation, 1–7. [70], Copyright (2019), with permission from Springer.

One promising option is based on intrinsic fluorescence or phosphorescence, i.e., photoluminescence of MPs, as described in Section 3.4. In such a case, the staining of MPs can be avoided. The potential challenge in in situ detection will be luminescence from organic materials, such as chlorophyll, that disturbs the luminescence from MPs similar to the case of Raman measurements. However, Ornik et al. [70] successfully demonstrated a photoluminescence detection technique (see Figure 10) using a high-power blue light-emitting laser to discriminate between plastic and non-plastic luminescence spectra. Additionally, the disturbance from organic materials may be handled by sophisticated chemometrics.

Moreover, MPs arriving in the aquatic environment may already have coloration due to color print on food and non-food plastic products based on the use of dyes and pigments [125]. The formulation of ink for plastic color print involves different types of resins, oils, and solvents such that ink may have spectral properties similar to those of plastics. Furthermore, each plastic usually requires its specific ink formulation, and thus provides a means to identify such colored MPs using possible fluorescence signals directly from the overlayer print. Moreover, the different spectral properties of the dyes and pigments of the prints, or the coloring agents of plastic without a print, can be exploited for the identification of the MPs.

**Figure 11:**
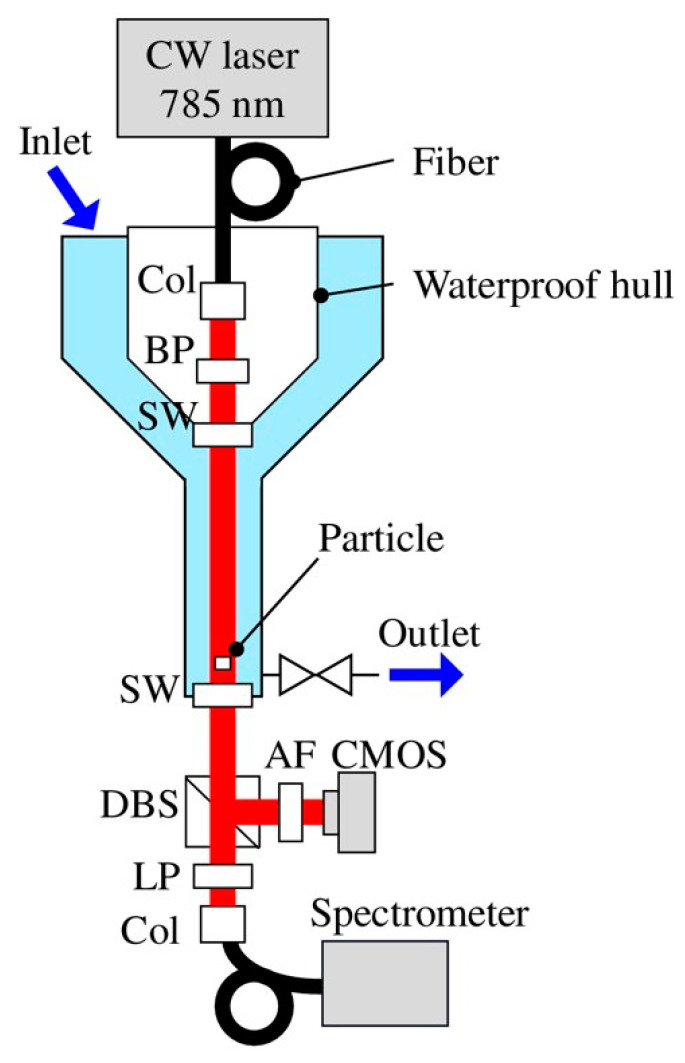
Experimental setup: Col, collimator; BP, bandpass filter; SW, sapphire window; DBS, dichroic beam splitter; AF, attenuation filter; and LP, long pass filter. Trapped particles are probed with holography and Raman techniques [130].

Another type of optical method that can be used for the identification of both MPs and organic particles to yield information on the size and thickness of the particles could be based on digital holography (DH) [126]. In this method, one uses the imaging principle of holography based on a coherent laser light source. An interference pattern generated by the interaction of light radiation transmitted through an object is captured on a CCD camera together with a reference radiation signal. Thereafter, mathematical data analysis is used to reconstruct the image of the microparticle. Merola et al. [127] studied the identification of MPs and microalgae using digital holography, albeit in a Petri dish. Digital holography can be applied in relatively clear waters for in situ and real-time detection of particulates, including MPs. For example, this has been demonstrated for in situ detection of plankton [128], and an algorithm to analyze the arbitrary shapes of micro objects, including fiber-type, has been given in the literature of DH microscopy [129]. Similarly, toward practical applications, holography, coupled to Raman spectroscopy, has been demonstrated in large water flow volume, although for relatively large MP detection [130]; see Figure 11.

While conventional microscopy exploits 2D imaging of the MPs and requires the particle to be in the focal plane of the imaging system, the probability of capturing a single MP in in situ detection is typically very low. On the contrary, DH provides 3D imaging holograms as a function of time and monitors particles out of the focal plane as well. This allows more flexibility because it is possible to monitor, in addition to the shapes of the MPs, their locations and velocities in a water volume, yielding information on the particle thickness and the continuously varying refractive index of the ambient medium [131]. The practical implementation of a DH system can be based on the use of a high-power laser diode as a point light source to create a divergent beam. Such a diverging source could replace the use of imaging lenses, hence simplifying the system. For fast particle tracking purposes, a pulsed laser source, having a pulse width of tens of nanoseconds, may be suitable as the light source.

In in situ measurements, one could also use single-mode optical fibers to guide both the object and reference radiations of the DH system into a measurement location of a suitable detector. The advantage of the single-mode fiber is its stability against mechanical disturbances such as movement in shallow waves and deep-water currents. Furthermore, the output ends will produce strongly diverging light beams and hence enable the monitoring of a large water volume. Already, the potential of DH in MP identification has again been demonstrated by combining it with Raman spectroscopy to identify the size and type of MPs in the laboratory [130].

Apart from using optical fibers in the digital holography, it can also be used for sensing applications or for guiding signals in shallow and deep water. For example, Kenny et al. detected phenols and gasoline in groundwater using laser-induced fluorescence along optical fibers [132]. This rather old technique can also be exploited for the detection of MPs in water. In particular, with the recent development of miniaturized high-power laser sources and sensitive detectors, fiber optic sensor units based on these devices may be convenient for underwater MP detection.

The immersion liquids method [133,134] can be exploited for in situ detection of MPs and NPs. By injecting suitable non-toxic water-soluble liquid into the detection compartment of underwater optical sensors, with controlled water flow, one can detect a change in transmittance both in clear and turbid waters. The transmittance changes as a function of the mismatch between the refractive index of the binary liquid mixture and that of different plastic particles. The nice feature of the immersion liquid method is its validity for the detection of both MPs and NPs with regular or irregular shapes. However, this method may be suitable as a complementary detection technique.

### 6.2. Hamaker’s Constant, Interferometry, Effective Medium, Scattering, and Stoke’s Parameters

Regarding the detection of small NPs, light scattering is negligible, and most of the methods described above are questionable. In the case single NP detection, for example, light scattering, in the Rayleigh domain, is weak for NPs of 1–10 nm in size. However, as NPs aggregate, resulting in a relatively large size, the probability of scattering is in the Mie domain and becomes higher; hence, it is easier to detect an aggregate by light scattering. The aggregation of the NPs—homogenous or heterogenous—is due to Van der Waals forces. Homogenous aggregation of NPs can lead to enhanced light scattering beneficial for their detection. Heterogenous aggregation, on the other hand, hinders the identification of the individual plastic types, especially when they show overlapping spectral features. The issue of NP aggregation is likely to be enhanced by the size of the probing source that can lead to a weighted spectrum from the aggregate.

Additionally, in an aquatic environment, one would expect NPs to be irregular, having nanoroughness and other surface coatings that further cause their optical properties to deviate from their macroscopic properties [34]. The effect of Van der Waal’s forces causing aggregation can be predicted from UV–Vis–IR spectral properties of macroscopic plastics based on both wavelength-dependent refractive index and the absorption coefficient of the plastics. In nanoplastics aggregation, the so-called Hamaker constant plays an important role. For example, the Derjaguin–Landau–Verwey–Overbeek (DLVO) theory, which exploits the Hamaker’s constant and depends on the plastic type of NP, the salinity, as well as pH of water, has been applied for the detection of spherical NPs in water and their aggregation [5,6]. Primarily, the Hamaker’s constant can be obtained by using the Kramers–Kronig analysis method and a wide electromagnetic spectrum in the calculation of dielectric properties of the NP–water system. Readers interested in Hamaker’s constant and progress in its calculation can find more information in the review article of Rosenholm et al. [135]. Additionally, the detection of sub-diffraction-limit MPs could also benefit from wide-field scanning correlation interferometric microscopy [136]. Indeed, on its own, the background-free interferometric detection method has been used to detect low-index particles in water down to 10 nm in radius [137]; coupled to other spectroscopic methods, this method may be a viable component of the integrated solution for in situ detection of NPs.

Further, to better understand the behavior of NPs in the complex environment, Bruggeman effective medium theory [138] can be exploited in the simulation of such an environment. Bruggeman’s model predicts change of spectral fingerprints of the NP–water system as a function of the concentration of NPs in water volume, but under the assumption of negligible scattering of the probe wave. The method is valid for real nanoparticles such as NPs in water. The change in the spectral properties of such a dilute liquid–solid mixture can be effectively predicted by this method.

Further to the developed optical methods in Section 4, the use of speckle analysis is also promising in screening MPs in situ. Analysis of speckle intensity can provide information on the scattering medium such as MPs in water, yielding parameters including the aggregation of MPs [139] in salty water, thickness [140], and surface roughness [141]. Additionally, the change of polarization state of the probe laser light or other light sources can be determined by observing the so-called Stokes parameters [142]. It becomes thus a powerful method to obtain information from the optical properties of MPs. Implementing this method, however, requires typically the use of two light polarizers, one after the light source and the other before the detector, in transmission mode. In reflection mode, both polarizers will be placed on the same side in the paths of the incident and the reflected light waves. The speckle contrast calculation and the fixed-wavelength transmittance measurements can be integrated into a portable sensor to study the sedimentation of MPs in a controlled environment. Similarly, modifying the systems of dynamic and multi-angle light scattering methods to include linear polarizers could lead to a more sensitive device to detect rod-like or other non-spherical particles. Brownian motion can cause rod-like MPs, for example, to rotate, which can be observed in the dynamic change of the scattered light pattern after passing through the polarizer. In principle, such a system with a complex dynamic speckle pattern could be analyzed, exploiting on time-dependent cross-correlation analysis of the speckle pattern to resolve sizes of scattering NPs and MPs, as suggested in [143].

## 7. Towards Full Integration

**Figure 12:**
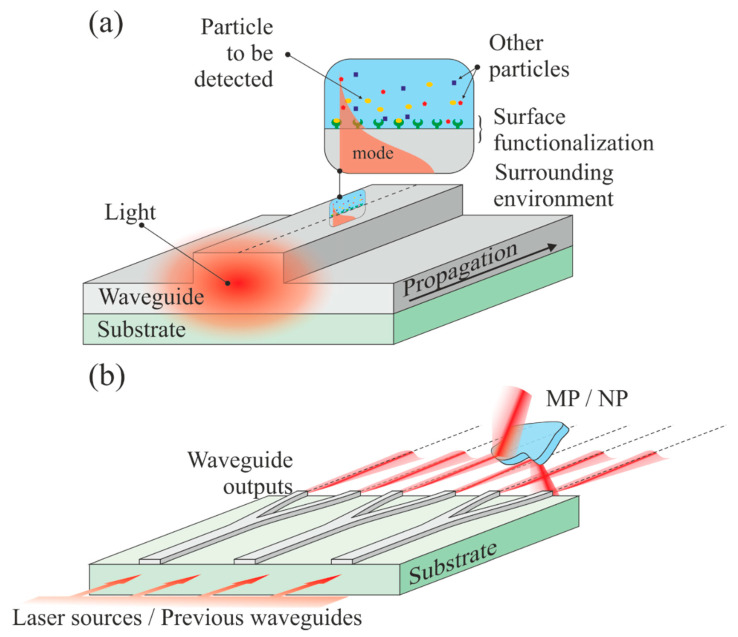
Waveguide-based sensors/detectors. (**a**) The top surface of a waveguide can be functionalized to attract specific molecules. The exponentially decaying portion of the propagating mode is interacting with the trapped particles, which yields a signal modification; (**b**) multiple channel waveguides illuminate a sample (which can be very large), which can reflect, deflect, or diffract the beams.

Moving a step further in the integration of optical methods requires the development of lab-on-chip [144] or lab-on-fiber [145]. A simplified vision of waveguide sensing provides two options, since the waveguide can be used either as a probe or as a source. In the first case, the sensing or detection mechanism is often evanescent [146,147]. However, specific waveguide configurations may allow interaction between the analyte and the core propagating light, such as in the cases of slot [148], and anti-resonant reflecting optical waveguide (ARROW)-based sensors [149]. In the second case, light emerging from the waveguide can be used to image, illuminate, and interact with the surrounding medium. The two mechanisms are illustrated in Figure 12. An obvious issue with such devices is the typical size of the MPs and NPs to be detected. A waveguide with an output mode diameter ranging from 200 nm (silicon nano-waveguides [150]) to a few micrometers (glass or ceramic waveguides [151]) is common. Fibers can provide modes with very large diameters depending on the core-size. Although the purpose here is not to provide a detailed review of waveguide sensing, we state a few facts that can be used to detect plastic particles at nearly all scales.

### 7.1. Evanescent Sensing

This type of sensing comes from the interaction of the exponentially decaying portion of a guided mode with the environment surrounding the waveguide (see Figure 11a). In such a case, the waveguide can be a channel, a slab, or a fiber. Numerous sensing schemes can be imagined from this configuration, and we refer the reader, for instance, to the following reviews [152,153,154,155]. The waveguide (core or cladding) can be chemically functionalized, i.e., labeled, to trap a specific group or molecule on the surface, which will modify the signal [156]. Instead of a particular chemistry, the surface can be patterned onto a nanostructure, leading, for instance, to fiber Bragg gratings that have demonstrated their power in sensing [157]. Such a sensing mechanism is often used for refractive index [158], fluorescence [159], and Raman/SERS [160] measurements. In the particular case of micro and nanoplastics, for which the concentration is extremely small, such a configuration is not suitable, although extreme sensitivity is possible.

### 7.2. Waveguide-to-Free Space Sensing

Channel waveguides on a chip offers numerous possibilities, especially if we consider a free-space output combined with an imaging lens system allowing the beams to propagate in free space in a controlled manner (focused, collimated). A simplified scheme is illustrated in Figure 11b. It is composed of a source (usually a coherent laser light) coupled to broad channel waveguides that are separated and ramified until they provide the desired amount of outputs. Each output may have a different wavelength or phase. In its simplest form, such a sensor is a Young interferometer, in which the two-point sources are the waveguide outputs from a Y-junction [161]. Arrayed waveguide grating (AWG) [162,163], light detecting and ranging (LiDAR) [164], and phased array (PHASAR) [165] are in constant development and are already in use for the monitoring of very large areas, such as forests. If LiDAR can be used for the detection and positioning in space, similarly to RADAR, the AWG system forms an excellent basis for spectrometry and is already extensively used in telecommunication systems for dense wavelength multiplexing. Fully integrated photonic circuits for LiDAR are used in automotives. Waveguide and fiber sensing are nowadays ubiquitous, since different physics, such as plasmonics [166], for instance, can be merged to raise the sensitivity of the device.

### 7.3. Waveguide-to-Free Space Sensing

Despite all these tools, so far no application of integrated optics has been devoted to the monitoring, detection, screening, or analysis of MPs and NPs. In the near future, it seems reasonable to expect to see the development of spectrometers, diffractometers, and speckle analyzers combined with artificial intelligence (AI) schemes for data analysis and processing for the detection in situ of micro and nanoplastics. Several key advances are paving the way. It has been recently demonstrated that fluorescence signals from microparticles can be detected even in mixtures [167]. Optical waveguides are ideal devices to be integrated with microfluidic channels, leading to a device capable of detecting antigens, DNA, µRNA, and so on [168,169]. Low power consumption, low weight, mass production compatibility, multi-physics, and chemistry compatibility are some advantages of waveguides over free-space solutions. The price to pay is the extremely challenging fabrication and high sensitivity to fabrication limitations that often make the devices not fully reliable.

In summary, to obtain development of future multi-purpose in situ optical solutions for comprehensive identification and quantification of MPs and NPs beneath water surfaces, a clever way of integrating miniature versions of FTIR, Raman, fluorometer, conventional spectrophotometer, and the novel methods described in this section seem viable options. Based on the similarities between conventional plastics and bioplastics [170], we believe that the methods described above are applicable to MPs and NPs originating from bioplastics. We note that for in situ applications, there are possible technical issues, such as contamination of optical elements of the probing window of the proposed sensor, that can arise. Nevertheless, this problem can be solved by exploiting micro and nanopatterning and ultrasound cleaning techniques. Smart sensors connected to AI for data processing based on integrated optics can be integrated into a water filtration membrane to yield real-time information on the presence of MPs and NPs captured by the membrane.

## 8. Conclusions

In this review, we have described a variety of different types of useful conventional but also novel optical methods to detect micro-and nanoplastics sampled from aquatic environments at the water surface, beneath the water surface, or embedded within it. The advantages and disadvantages of the individual traditional photonics-based solutions such as FTIR, Raman, and hyperspectral methods and their application to real microplastics obtained from the municipal wastewater system were also discussed. It is evident that FTIR and Raman techniques play a key role in the identification of MPs harvested from the environment. Portable and remote sensing devices based on FTIR, Raman, speckle metrology, and hyperspectral imaging and their potential for MP detection were also highlighted. Furthermore, novel and emerging optical methods such as digital holography, photoluminescence, and optical waveguides were proposed as useful and promising future techniques for in situ identification of micro and nanoplastics in complex aquatic environments. We conclude that the development of a reliable and multifunctional device for the accurate detection and monitoring of MPs/NPs in situ certainly could benefit from the intelligent integration of photonics-based solutions. Indeed, such a study is ongoing where some of the proposed novel methods are coupled together for the identification of MPs in water.

## Data Availability

The data presented in this study are available on request from the corresponding author.
